# Lifestyle shapes preclinical social and microglial deficits in an Alzheimer’s disease mouse model

**DOI:** 10.1038/s41380-025-03368-4

**Published:** 2025-12-12

**Authors:** Fanny Ehret, Birte Doludda, Hang Liu, Sindi Nexhipi, Hao Huang, Fabian Rost, Rupert W. Overall, Warsha Barde, Annette E. Rünker, Michael Sieweke, Andreas Dahl, Mirko H. H. Schmidt, Gerd Kempermann

**Affiliations:** 1 Institute of Anatomy, TU Dresden, 01307 Dresden, Germany; 2 DZNE – German Center for Neurodegenerative Diseases, 01307 Dresden, Germany; 3 CRTD – Center for Regenerative Therapies, TU Dresden, 01307 Dresden, Germany; 4 OncoRay – National Center for Radiation Research in Oncology, Faculty of Medicine and University Hospital Carl Gustav Carus, TU Dresden, 01307 Dresden, Germany; 5 German Cancer Consortium (DKTK), Partner Site Dresden, and German Cancer Research Center (DKFZ), 01307 Dresden, Germany; 6 Helmholtz-Zentrum Dresden-Rossendorf (HZDR), Institute of Radiooncology – OncoRay, 01328 Dresden, Germany; 7 University of Bonn, Life & Medical Sciences Institute (LIMES), 53115 Bonn, Germany; 8 DRESDEN-Concept Genome Center, Center for Molecular and Cellular Bioengineering (CMCB), TU Dresden, 01307 Dresden, Germany; 9 Institute of Biology, Humboldt University, 10099 Berlin, Germany

**Keywords:** Neuroscience, Cell biology, Stem cells

## Abstract

Alzheimer’s disease has a long preclinical phase, during which no overt signs of the manifest disease are present, but subtle, usually non-specific changes are already detectable. Emerging early biomarkers underscore the importance of this phase for preventive measures including lifestyle interventions. As a reductionistic model for lifestyle factors, we used a novel enrichment paradigm in which App^NL-G-F^ knock-in mice were continuously tracked until 7 months of age. Despite minimal plaque burden and no memory impairment at that age, there were early and progressive deficits in social parameters — such as following behavior, social interaction, and exploration – suggesting preclinical behavioral vulnerability. Altered correlations between adult neurogenesis and social parameters linked neural plasticity to preclinical behavior. Plasma profiling at 3 months identified early systemic shifts in markers of inflammation and apoptosis that predicted later cortical pathology. We found increased microglia coverage in more socially active animals. More actively exploring controls, but not App^NL-G-F^ mice, exhibited more ramified and less amoeboid microglia, suggesting that AD pathology impairs immune surveillance at a very early stage. Single-cell RNA sequencing of hippocampal microglia revealed that enrichment dampened interferon-responsive microglia, which typically increase as amyloidosis advances. A shifted immune response was also measured by reduced transcripts related to antigen processing and presentation and by increased chemokine signaling. Our study demonstrates that the preclinical phase of AD is not silent, but even in a reductionistic knock-in model characterized by early interwoven preclinical changes in multiple domains, including brain plasticity, behavioral trajectories, sociality and immunity.

## Introduction

Alzheimer’s disease (AD) is characterized by a silent preclinical phase (referred to as pre-AD), in the course of which not only biomarkers increasingly predict the risk of disease manifestation but also subtle, usually non-specific behavioral changes become detectable [1–3]. This preclinical stage is considered the critical target for the development of successful preventive strategies, including but not limited to prevention through lifestyle interventions [4, 5].

Onset and course of AD are shaped by the multi-faceted interplay between genetic susceptibility, environmental factors, and the complex mix of behaviors that are commonly subsumed under lifestyle [6]. Addressing modifiable risk factors through lifestyle has been estimated to potentially allow the prevention or delay of up to 40% of dementia cases in the future [7]. Among them, physical inactivity and low educational attainment are the highest morbidity risk factors [7, 8].

WHO and The Lancet have published lists of key preventive lifestyle factors that have found their way in widely distributed recommendations for the prevention of AD [7, 9]. Derived from large epidemiological studies, these lists focus on quantifiable, usually physical factors, but neglect many other aspects, for which the scientific evidence is lower. Among these risk factors are loneliness and social isolation, which have also been found relevant for AD progression particularly at the preclinical stage [10]. A large number of other potentially effective, yet vastly varying lifestyle factors with high face validity have been identified [11], raising the question of the common biological denominators of lifestyle. These principles are challenging to assess in sporadic AD, particularly during the clinical silent stage when prevention would be most effective. Additionally, inter-individual variation in response complicates the development of effective primary and secondary prevention strategies. In contexts of high genetic background variation, as common in humans, the specific behavioral impact on variance cannot be discerned. This variability is also dynamic and, as animal studies reveal, emerges even in cases of a fixed pathological genotype [12].

In a knock-in animal model of AD, we discovered that subtle behavioral changes emerged at the preclinical stage, long before disease-specific deficits. Even during this disease-free period, the AD mice showed reduced habituation to their environment and less inter-individual variation in behavior, which was less predictable, but paradoxically also less flexible [12]. This observation with its relevance for our understanding of preclinical AD stages and prevention during this phase called for a deeper, more mechanistic analysis.

At the behavioral level, we focused on social interaction. In humans social interaction contributes to robust cognitive reserves [13, 14], but in elderly people can become limited by an increasingly rigid and narrower range of general activity [15, 16]. At the structural level, we focused on the hippocampus, which is prominently affected by AD. Adult hippocampal neurogenesis, as a particular type of plasticity, is dramatically reduced in late AD [17]. It is necessary to form stable behavioral trajectories [18] and there is a growing literature on its role in social behavior [17, 19, 20]. Moreover, the plasticity influenced by adult neurogenesis, which is dependent on activity and experience, appears to drive the individualization of the hippocampal circuitry [11].

At the mechanistic level, we followed ideas developed by Amanda Sierra and colleagues, who proposed that microglia present a central regulatory link between environment, lifestyle, and adult neurogenesis [21].

The interest in neuroinflammation and microglial activity as modifiable features of AD pathology has grown, especially as genetic studies identified AD risk variants, such as TREM2 [22–24]. However, to which extent the brain’s immune surveillance system with its established involvement in the course of AD, might be influenced by early behavioral intervention has not been addressed in long-term ENR experiments. Whereas the role of innate and adaptive immunity in AD is relatively well-established, the exploration of behavior-related modulation of immune factors and microglia in the formation of reserves and the progression of AD remain uncharted territory. We hypothesized that microglia would already show an altered response to changes in environment, behavioral activity, and social interaction in otherwise healthy mice with a genetic predisposition for AD.

To address this idea, we used the App^NL-G-F^ mouse model (referred to as NL-G-F) that carry the Swedish, Bayreuth/Iberian and Artic mutations and show a mild onset in pathology starting around three months, but no clinical symptoms by conventional standards. Also, the end point of our experiment at 7 months of age was chosen such that it would precede the onset of overt clinical symptoms [25]. While our previous study was based on the NL-F model, the NL-G-F model has also been used by others to predict AD at pre-symptomatic stages with an AI-based classifier. That study found subtle behavioral changes starting at age 8–12 months, which is also still ahead of the onset of cognitive impairments [26]. We predicted that changes in behavior, plasticity, and immunity would be detectable with our paradigm at an even earlier preclinical stage.

Thus, the mice were kept in a large ENR enclosure, consisting of 70 connected cages [27]. Radiofrequency identification (RFID) based tracking of individual mice allowed us to capture their activity patterns and social interactions. The initial immune cell response was assessed from blood samples at 3 months. Microglia response and adult neurogenesis were evaluated histologically at 7 months after 5.5 months under complex enrichment. In addition, single cell-RNA (sc-RNA) sequencing of microglia was performed to explore molecular mechanisms through which enrichment may impact microglia function in AD.

## Materials and methods

### Animal husbandry

All animal husbandry and experiments were in accordance with European and national regulations and approved by the local authority (Landesdirektion Sachsen; TVV 28/2020). App^NL-G-F/NL-G-F^ and App^NL/NL^ mice were obtained from Riken Institute containing a Swedish (KM670/671NL), Arctic (E693G) and Beyreuther/Iberian (I716F) mutation [28]. Mice were maintained on a 12 h light/dark cycle with food and water provided *ad libitum*. At 4 weeks, animals were randomly assigned to either standard housing (STD) or enriched housing (ENR) in custom-built multi-cage system (PhenoSys GmbH, Berlin, Germany; now marketed as “ColonyRack”). The experimental groups consisted of 33 female mice per genotype in ENR with all 66 mice housed together, and 19 mice per genotype in STD with groups of 3–4 mice separated by genotype. At 5 weeks, a glass-coated microtransponders (SID 102/A/2; Euro I.D.) was injected subcutaneously into the neck of ENR mice under anesthesia, followed by ENR entry one week later. To maintain novelty of the ENR, the equipment, i.e. toys and huts, was changed biweekly in terms of position and complexity. Locations of food and water remained unchanged. At 13 weeks, mice were injected 3x with IdU (57,5 mg/kg) and blood samples were taken from vena facialis. At 28 weeks, all mice were removed from the ColonyRack. For microglia isolation and scRNA Seq, mice (5 ENR and 3 STD mice per genotype) were killed by cervical dislocation.

Animals for histological analysis (28 ENR and 16 STD mice from each genotype) were killed with a mixture of ketamine/xylazine and transcardial perfusion with 4% paraformaldehyde (PFA). Final blood samples were taken from the heart (right atrium) prior to perfusion. Brains were left in 4% PFA over night at 4 °C and transferred to 30% sucrose before sectioning.

### Behavioral analysis based on tracking data

Contact of chip-tagged mice with radio-frequency identification antennas, located at connecting tubes of the ColonyRack, were recorded using the software PhenoSoft Control (PhenoSys GmbH), which saved antenna and mouse identifiers together with the time stamp into a database. The raw data reduced to 5 s intervals and additional meta data can be provided on request.

Calculation of roaming entropy (RE) was performed as previously described [29, 30]. As mice are nocturnal animals, only the events recorded during the dark phase were retained. Cumulative RE (cRE) was calculated by cumulative addition of mean RE from the 12 time-blocks.

The social scores (social distance, following events) were calculated using the Rpackage ColonyTrack (see https://rupertoverall.net/ColonyTrack/index.html). In brief, the social distance score is the average shortest distance between a target mouse and all other mice per day. A following event is recorded when one mouse triggers an antenna together with another mouse within <1 s, traveling in the same direction. The total number of events is reported for each mouse for each day.

### Analysis of blood samples and ELISA protein detection

Blood was collected into EDTA-coated microvettes (Sarstedt), plasma was separated by centrifugation, sent to OLINK (Upsala, Sweden) and analyzed by proximity extension assay (PEA) for 96 proteins. Quality controls and data normalization was performed by Olink [31]. Provided normalized protein expression (NPX) values on a log 2 scale were used for comparisons.

Serum samples taken at 7 months were used to measure Casp3 (E-El-M0238, Elabscience) and Ill1α (EA100140, Origene) protein levels by colorimetric sandwich ELISA following manufactures instruction.

### Histology

Tissue fixation and immunohistochemistry adult neurogenesis analysis were performed as previously described [12]. For the detection of plaques, sections were incubated with X-34; for details see Supplementary S1.

Fluorescent staining of Iba1, Ki67, Trem2 and Draq5 was performed to detect and phenotype microglia. For details see Supplementary S1 and for validation of automatic segmentation Supplementary Fig. 2. For the detection of IdU^+^- and Dcx^+^-cells the peroxidase method was applied on every 6^th^ section. For details see Supplementary S1.

### Sample processing for sc-RNA-seq analysis

Hippocampal tissue was mechanically dissociated and microglia was sorted by FACS as shown in Fig. 5A. For details see Supplementary S1.

### Statistics

Experiments were carried out with the experimenter blinded regarding the experimental group. Statistical analyses were done using the software Prism 9 (GraphPad) and R (v4.1.2; R Core Team 2021); for details see Supplementary S1 & T2 for statistics description.

## Results

### Reduced social engagement and habituation behavior in NL-G-F mice at pre-symptomatic stages

**Fig. 1:**
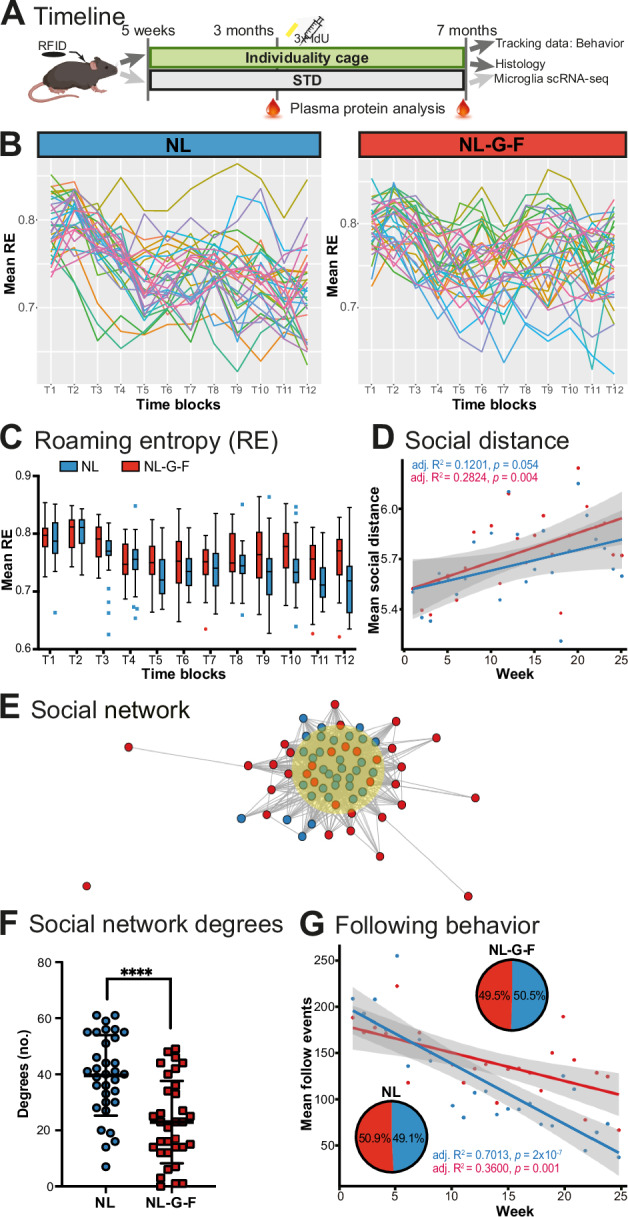
Deficits in exploratory activity and social engagement in NL-G-F mice. **A** Experimental timeline outlines injections, entry into cage system, plasma collection and analyzed parameter. **B** Individual exploration trajectories plotted as RE over consecutive time blocks (2 weeks each). **C** Mean RE per time block, depicted as box plots, indicating reduced RE over time in NL, but not in NL-G-F mice. Center line - median; upper and lower hinges - first and third quartiles; whiskers: Tukey. Repeated measures ANOVA with effect on genotype *p* > *0.0001*, time and interaction *p* > *0.0001*. **D** Social interaction analysis based on mean social distance over time shows an increasing social isolation of NL-G-F mice. Linear regression parameters are shown. **E** Social network analysis on the basis of social distance depicts that more NL and fewer NL-G-F mice are in the center of the network. NL-G-F mice had fewer close social contacts. **F** Analysis of network degrees revealed a significant difference between NL and NL-G-F; unpaired t-test p < 0.0001; line at mean +/- SD. **G** Following events over time shown as linear regression. NL-G-F mice habituate their following behavior only insufficiently compared to NL controls. Pie charts depict genetic preference of following behavior; no preference was identified. ENR, enriched environment; ldU, Iododeoxyuridine; RE, roaming entropy; RFID, radiofrequency identification; STD, Standard housing. Significant differences are shown as ****p < 0.0001.

We co-housed a cohort of NL control mice carrying a non-pathological Swedish App mutation and NL-G-F mice additionally harboring the disease-promoting Beyreuther/ Iberian and Arctic App mutations in a complex enriched environment in their adult presymptomatic phase from 5 weeks to 7 months (Fig. 1A). Various behavioral parameters were derived from RFID-based tracking data of individual mice. Using roaming entropy (RE), a measure of the exploratory activity or territorial coverage [30], NL mice showed habituation to the environment over time, as demonstrated previously [12]. This habituation was attenuated in NL-G-F mice (Fig. 1B, C). Analysis of social behaviors revealed differences in following behavior, which over time decreased significantly less in NL-G-F mice than in NL controls (Fig. 1G). Neither genotype showed a preference for following a particular genotype (pie charts in Fig. 1G). Furthermore, over time NL-G-F mice increased their social distance more than NL controls (Fig. 1D). This is also captured in the social network derived from this parameter (Fig. 1E) and consequently in significantly lower degrees of interaction in NL-G-F mice (Fig. 1F). Thus, NL-G-F mice appear to have difficulties habituating to their environment, show lower social engagement, and spend more social time in following mates, which might indicate aversive or dominant behavior [32]. These characteristics are detected at a presymptomatic stage and might thus be the earliest behavioral signs of AD.

### The association between behavior and adult neurogenesis is disturbed in pre-AD

**Fig. 2:**
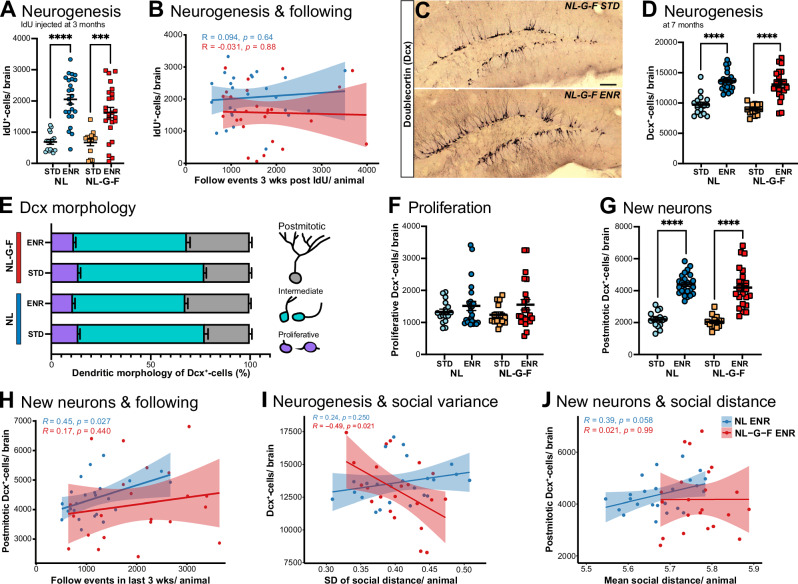
Adult hippocampal neurogenesis correlates with aspects of social behavior. **A** Quantification of neurogenesis by injection of thymidine analog IdU at 3 months of age. IdU^+^-cell counts were increased in both genotypes after ENR; two-way ANOVA effect of housing F_(1,77)_ = 57.81, *p* < *0.0001*, no significant effect of genotype or interaction effect. The variance was also increased by ENR, as measured by Brown-Forthsythe, *p* < *0.0001*. **B** Pearson correlation revealed no correlation between neurogenesis and behavior at this stage. **C** Micrographs depicting the difference in Doublecortin (Dcx) expression in STD vs. ENR housed NL-G-F mice. Scale bar 100 µm. **D** Number of Dcx^+^-cells analyzed at 7 months is higher in ENR compared to STD housing, two-way ANOVA F_(1,73)_ = 90.85. Effect on variance STD vs ENR, Brown-Forthsythe *p* < *0.0001*. **E** Classification of Dcx^+^-cells into proliferative, intermediate and postmitotic states based on morphology. For each group the cell states were plotted as stacked bar graph. **F**, **G** Quantification of total numbers of Dcx^+^-subtypes showed no effect of ENR on proliferative but on postmitotic stages, F_(1,73)_ = 127.3 *p* < *0.0001*. **H** Postmitotic Dcx^+^-cell counts correlated with the following behavior of the last 3 weeks in ENR. Controls, but not NL-G-F, showed significant interaction, as shown by Pearson’s R. **I** The interaction between the standard deviation (SD) for social distance over the entire ENR period and Dcx^+^-cell counts for individual animals are presented including Spearman’s R. **J** The mean of social distance in correlation to the number of postmitotic Dcx^+^-cells, Spearman’s Rs are shown. Significant differences are shown as ***p < *0.001;* ns, non-significant. STD standard housing, ENR enriched environment, IdU iododeoxyuridine.

To determine potential links between behavior and brain plasticity in terms of adult neurogenesis, we administered IdU to 13-week-old mice, after the first 8 weeks of ENR-housing. When assessed at the end of the experiment, IdU^+^-cell counts – reflecting surviving cells born at 13 weeks – were increased in NL and NL-G-F mice in ENR compared to STD (Fig. 2A), but the increase was attenuated in NL-G-F mice. We here found no correlation of neurogenesis with following behavior (Fig. 2B), or other social and exploratory scores (Supplementary Fig. 1 E, F) for either genotype, possibly due to the relatively early labeling time-point.

We further evaluated the number of newly generated, neuronally committed (Dcx^+^) cells at the end of the experiment (Fig. 2C). Although we did not observe any genotype-effects on in Dcx^+^-cell counts, a robust effect of ENR on Dcx+ counts persisted in NL-G-F mice even at this very early pathological stage (Fig. 2D). We classified the Dcx+ cells into proliferative, intermediate, and postmitotic subtypes based on their morphology (Fig. 2E-G). The ENR effect was specific to postmitotic neurons for both genotypes. However, in NL controls, the number of postmitotic Dcx^+^-cells correlated positively with the counts for following behavior during the final 3 weeks of ENR. This relationship was blunted in NL-G-F mice (Fig. 2H). Furthermore, in control mice, higher counts of postmitotic Dcx^+^-cells were associated (as a trend) with higher means (Fig. 2J) and variances (SD; Fig. 2I) in social distance. The opposite was true for NL-G-F mice (Fig. 2I, J). In conclusion, our findings underscore an association between social and exploratory behavior and adult neurogenesis that is disturbed in early preclinical stages of AD.

### Unveiling the interplay of plasma proteins, behavioral activity, and plaque pathology

**Fig. 3:**
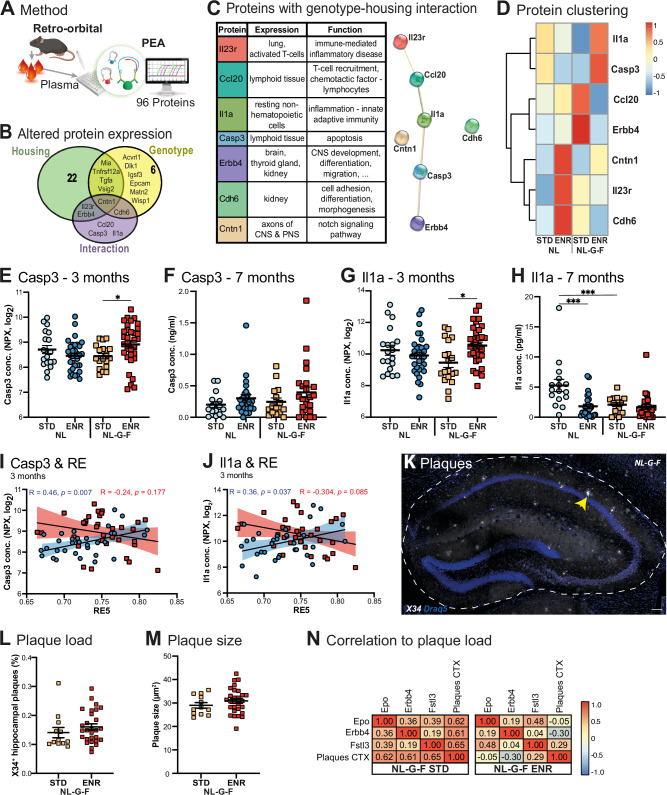
Analysis of plasma at 3 months identifies proteins relevant for inflammation, apoptosis and plaque pathology. **A** Illustration of the methods used to isolate and analyze plasma samples at 3 months, i.e., after 8 weeks of experimental housing. **B** Venn diagram of proteins with a significant effect for the different parameters (housing, genotype and interaction of both). **C** Illustrates the expression of the 8 proteins with significant genotype-housing interaction across the human body. **D** Spearman correlation matrix with hierarchical clustering of the proteins with interaction effect, depicting relative expression under the different conditions. **E** Protein expression of Caspase 3 (Casp3) at 3 months measured by PEA revealed a significant interaction effect, two-way ANOVA F_(1100)_ = 6.718, *p* = 0.011, with STD vs ENR in NL-G-F, *p* = *0.036*; *N* = 19 for each STD cohort and *N* = 33 for each ENR cohort on all PEA assays. **F** Casp3 levels at 7 months measured by ELISA (**G**) Concentration of Il1a measured by PEA at 3 months with a significant interaction effect, F_(1100)_ = 6.706, *p* = *0.0079*, with STD vs ENR in NL-G-F, *p* = *0.025*. **H** Il1a concentration at 7 months measured by ELISA did reveal a significant effects of genotype F_(1,84)_ = 9.829 p = 0.0024, housing F_(1,84)_ = 13,54 *p* = *0.0004* and interaction F_(1,84)_ = 8.505 *p = 0.0045*; with STD vs ENR in NL *p < 0.0001* and STD NL vs STD NL-G-F *p < 0.0001*. **I, J** Correlation analysis between Casp3 concentration (**I**) or Il1a concentration (**J**) at 3 months and roaming entropy (RE) of the corresponding time block (10 days). Pearson’s Rs for the two genotypes are shown. **K**–**M** Plaque load and size in the hippocampus was analyzed by X34, a derivate of Congo red. **K** Representative micrograph used for segmentation of plaques, a large dense plaque is marked with yellow arrow. Neither plaque coverage (**L**) nor plaque size (**M**) was influenced by housing condition. **N** Correlation matrix of regulated proteins with significant correlation to plaque load in the cortex as depicted by color code. Plotted values are Pearson’s Rs. Significant differences are shown as *p< 0.05, **p < 0.01, ****p < 0.0001. ELISA enzyme-linked immunosorbent assay, PEA proximity extension assay, Con concentration, CTX cortex, STD standard housing, ENR enriched environment. Scale bar in K, 50 µm.

To investigate potential early biomarkers for pre-AD stages that are modifiable by lifestyle factors, we analyzed 96 plasma proteins at 3 months of age (Fig. 3A). Of these, 29 proteins were altered by housing, 12 by genotype (Fig. 3B), and 7 proteins (Il1a, Casp3, Ccl20, Il23r, Erbb4, Cntn1, Cdh6) displayed a genotype-housing interaction (Fig. 3C, D). These 7 proteins are relevant for diverse organs beyond the central nervous system (Fig. 3C) and, as evaluated by String analysis, play central roles in receptor signaling pathways, inflammation, apoptosis, and neuronal differentiation. We investigated two candidate proteins in more detail: Casp3, a mediator of apoptosis, and Il1a, an inflammation-related cytokine. Both were specifically increased in NL-G-F mice housed in ENR and slightly reduced in STD housing compared to NL controls (Fig. 3D, E, G). However, this positive regulation was no longer evident at 7 months (Fig. 3F, H). Interestingly, at 3 months the plasma levels of both proteins correlated positively with behavioral exploration (RE of time block 5; Fig. 3I, J) in NL controls. This correlation was absent (Casp3; Fig. 3J) or almost inverted (Il1; Fig. 3J) in NL-G-F mice. None of the investigated proteins showed a correlation with parameters of social behavior (Supplementary Fig. 1 A, B).

We further explored potential correlations between the identified 7 proteins and AD pathology (housing groups of NL-G-F mice) assessed using X34 for labeling and quantification of plaques. ErbB4 levels were elevated in NL-G-F mice in STD but remained normal in ENR (Supplementary Fig. 1 C) and correlated with the cortical plaques in STD but not ENR (Fig. 3N), although neither plaque load nor size differed between STD and ENR (Fig. 3L-M, Supplementary Fig. 2 H, I). Moreover, a weaker correlation of Erbb4 with exploratory behavior dependent on the genotype was found (RE5; Supplementary Fig. 1 D). In addition to the proteins with genotype-housing interaction, the levels of two further proteins, Epo and Fstl3, correlated positively with cortical plaque load in STD-housed NL-G-F mice; this correlation appeared weakened or even showed the opposite trend in ENR (Fig. 3N). The data suggest that these proteins might be relevant for AD pathology and that this interplay can be modulated by ENR, at least at pre-AD stages.

### Microglia function in response to behavior and pathology

**Fig. 4:**
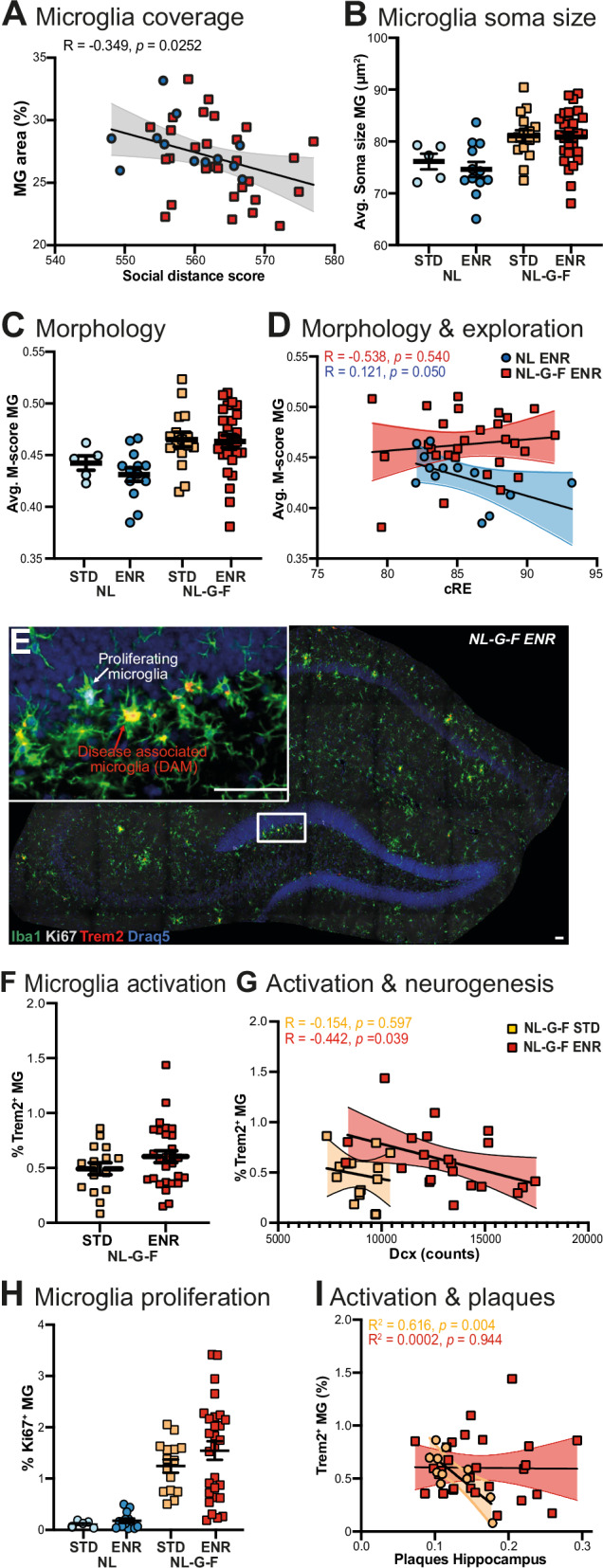
Histological microglia and plaque analyses in the hippocampus implicate microglia as mediator of environment x plasticity interaction. **A** Automated segmentation of Iba1^+^-microglia (MG) revealed correlation between MG area and social distance independent of genotype, Pearson’s R is presented. **B** An effect of genotype on soma size was detected, two-way ANOVA F _(1,58)_ = 14.22 NL vs NL-G-F *p* = *0.0004*. **C** Analysis of MG morphology using the M-score (a quantitative metric for microglial morphology along the ‘ramified’ to ‘amoeboid’ spectrum based on cell size and roundness) revealed an effect of genotype but not of housing condition, two-way ANOVA F_(1,58)_ = 10.02, NL vs NL-G-F *p* = *0.0025*. **D** Correlation of the cumulative explorative activity (cRE) and M-score; Pearson’s R identifies a significant relation only in controls. **E** Representative micrograph used for automated segmentation of microglia based on Iba1, proliferation marker Ki67, and activation marker Trem2. **F** Disease activation of microglia by Trem2 expression was not influenced by housing conditions. **G** In NL-G-F mice, Trem-2 dependent MG activation correlated with amount of Dcx^+^ neuronal cells in ENR but not STD conditions as evidenced by Pearson’s R. **H** The percentage of proliferative MG is increased in NL-G-F compared to NL mice, two-way ANOVA F_(1,58)_ = 32.79, *p* < *0.0001*; a further increased variance was seen upon ENR housing, Brown-Forsythe test *p* < *0.0001*. **I** The percentage of Trem2-dependent MG activation in the hippocampus correlated with the plaque load in STD mice; this interaction was abolished in ENR mice. ENR enriched housing, MG microglia, STD standard housing, cRE cumulative roaming entropy. Scale bar in E, 50 µm.

We performed semi-automated image analysis to examine Iba1^+^-microglia morphology, proliferation (Ki67), and activation (Trem2) in the hippocampus (Fig. 4) and cortex (Supplementary Fig. 2 G-O). Since exploration and social engagement were affected in our AD model, it was remarkable that, regardless of genotype, there was a correlation between social distance and microglia coverage in the hippocampus (Fig. 4A) but not the cortex (Supplementary Fig. 2 O). This indicates a specific significance of hippocampal microglia in relation to social behavior. In neurodegenerative diseases, microglia transition to a state of disease-associated microglia (DAM) with enlarged soma, amoeboid morphology, and reduced ramification. Microglia of NL-G-F had larger somas (Fig. 4B) and more ramifications, based on the M-score [33] (Fig. 4C), regardless of housing condition. A smaller M-score is indicative of resting, ramified microglia, while a larger M-score reflects phagocytic, amoeboid microglia. Notably, a significant correlation between M-score and cRE was observed in NL controls, but not in NL-G-F mice (Fig. 4D). This indicates that the interplay between behavioral activity and microglia function might be disturbed in pre-AD, possibly because activated microglia change or lose their response to neuronal activity.

In addition, microglia proliferation was higher in NL-G-F than NL mice, which was not altered by ENR. Instead, ENR amplified the variance of proliferation (Fig. 4H). In NL-G-F mice, no significant difference in Trem2-dependent activation was observed between STD and ENR (Fig. 4F). However, the numbers of Trem2^+^-cells and newborn neurons were negatively correlated exclusively in ENR (Fig. 4G). NL-G-F mice show higher variances of proliferative microglia (Fig. 4H) and Dcx^+^-cells (Fig. 3D, F, G) in ENR compared to STD. Thus, ENR seems to expand the variances sufficiently to illustrate this connection, thereby amplifying individual differences.

Since microglia modulate neurogenesis by phagocytosing apoptotic cells [34], a shift towards increased Trem2-activation and consequently, inflammatory responses might negatively impact on adult neurogenesis. The fact that adult neurogenesis was not impaired between genotypes might be due to the early stages of pathology investigated. Despite similar plaque levels and sizes in the hippocampus and cortex between STD and ENR (Fig. 3L-M; Supplementary Fig. 2H, I), a correlation with microglia activation was identified in STD but not ENR (Supplementary Figs. 2D and F3). Overall, the data suggest that microglia play a mediating role for an individualized immune response by adjusting microglia morphology, proliferative activity, and activation status in response to behavior, pathology, and their interplay.

### Microglia response to ENR: Identification of cellular and molecular changes

**Fig. 5:**
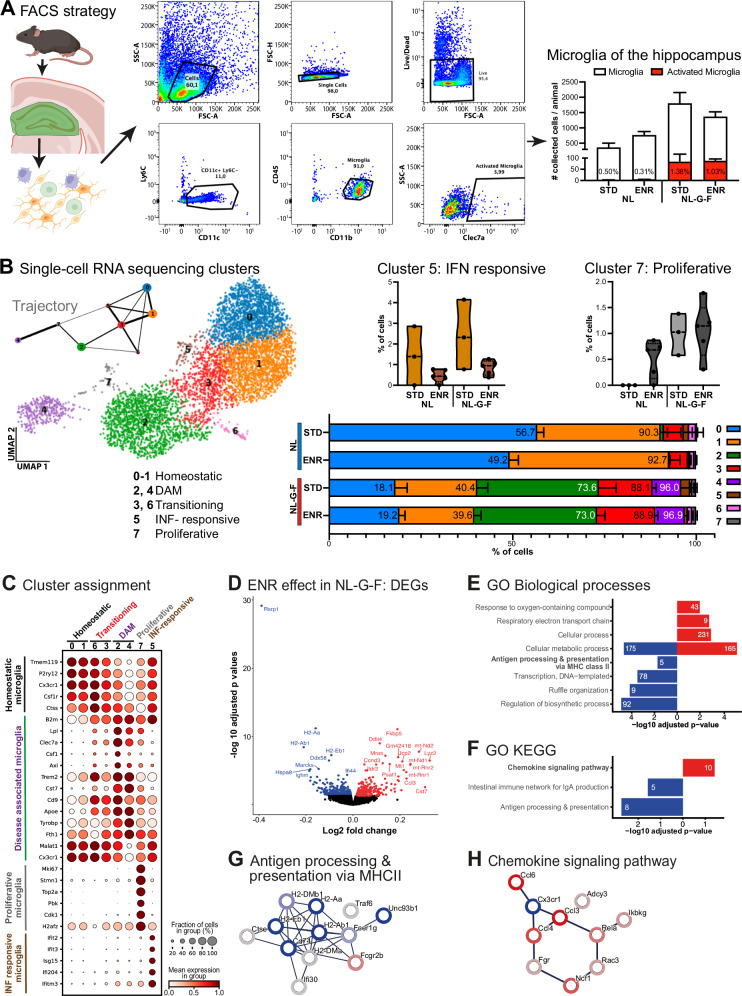
Sc-RNA sequencing of hippocampal microglia reveals cellular shifts and gene expression changes by ENR. **A** Illustration of the applied method and gating strategy to isolate microglia from hippocampal tissue. CD11c, Ly6C, CD45, CD11b were used as markers to sort all microglia; Clec7a was used to quantify the activation state. The percentage of activated microglia in the different groups is shown in the bar graph. **B** Sequencing of hippocampal microglia isolated as shown in A from the different conditions (from each genotype: *N* = 5 for ENR, *N* = 3 for STD). In total 5823 cells were analyzed. Seven clusters were identified, as shown on UMAP. The assignment of the clusters to cell states, as shown in the legend, was verified by marker genes, see **C**. Trajectory analysis (top left) of the identified clusters to analyze the continuum of state changes within this dataset was done using PAGA. The proportions of microglia subtypes according to the assigned clusters are shown as stacked-bar graph for each group. The small clusters 5 (interferon-responsive microglia) and 7 (proliferative microglia) showed a relevant regulation by ENR. **C** Dot plot depicting gene expression in the different clusters (cluster number indicated at the top). Each row shows the expression level of a selected key gene. The color represents the scaled expression changes. The size of the dots represents the fraction of cells expressing the gene in a given cluster. **D** Volcano plot of the ENR effect on DEGs in NL-G-F mice; significant hits were color coded for down regulation (blue) and up regulation (red). **E, F** Identified DEGs were analyzed by Gene ontology (GO) for biological processes (**E**) and KEGG pathways (**F**). Selected significant terms were plotted as bar graph and color coded for down (blue) and up (red) regulated processes or pathways; the number of significantly altered genes are given inside the bar. **G, H** Using STRING with ranks, two relevant protein networks identified in **E** and **F** were mapped with color coded halo based on log-fold change (FC). Edges represent protein-protein interaction. DAM disease associated microglia, FACS fluorescence activated cell sorting, INF interferon, ENR enriched environment, KEGG Kyoto encyclopedia of genes and genomes, MHC major histocompatibility complex, INF interferon, STD standard housing.

To gain a more comprehensive understanding of the ENR effects on the cellular and molecular signatures of hippocampal microglia at 7 months, we conducted sc-RNA sequencing of FACS-sorted microglia (Ly6C^-^ CD45^low^ CD11b^+^) including those in a proinflammatory state of neurodegeneration (Clec7a/Dectin-1^+^; Fig. 5A) [35]. Consistent with the histological findings, increased microglial presence was observed in NL-G-F mice compared to NL controls. Microglial activation was seen exclusively in NL-G-F mice, with no difference between the two housing conditions. Of the 16 mice used for hippocampal microglia isolation, 5823 microglial cells were analyzed in the sc-RNA transcriptome analysis (distribution see Fig. 5A). Unsupervised Leiden clustering identified seven distinct subclusters characterized by unique gene expression profiles (Fig. 5B-C). The assignment of these clusters to cellular phenotypes was based on published gene expression profiles [36, 37] (Fig. 5C). Compositional analysis (Fig. 5B) revealed a reduction in homeostatic phenotypes [0–1] in NL-G-F mice, accompanied by a shift towards activated DAM stages [2, 4].

Two clusters are of importance. First, interferon (INF)-responsive microglia [5] of both genotypes were diminished in response to ENR, suggesting a reduced inflammatory microenvironment. Second, the proliferative cluster [7] confirmed the histological data of heightened proliferative activity of microglia in NL-G-F and increased variance of this parameter for both genotypes in ENR. These observations indicate that although ENR has no effect on DAM activation per se, it has a positive impact on distinct microglial subsets and inflammatory activation. Trajectory inference (Fig. 5B) predicts that the majority of proliferative cells derive from or transition into DAM clusters, which develop primarily from homeostatic and transitioning microglia as reported previously [36]. INF-responsive microglia, however, do not transition directly from DAM clusters, but rather from homeostatic microglia.

Analysis of differentially expressed genes (DEGs) between housing conditions revealed intriguing potential targets in NL-G-F mice (Fig. 5D-H). Although a focused examination of cluster 5 or 7 was limited by low cell numbers, Ifi204 was notably upregulated in cluster 5 after ENR (adjusted *p* = *0.023*). This protein plays an important role in innate immunity [38, 39], as it is relevant for the production of inflammatory cytokines (DEGs are provided as Supplementary T1). Subsequently, we focused on DEGs between STD and ENR of NL-G-F mice in all clusters (Fig. 5D). Notably, ENR had a favorable influence on genes in Gene ontology (GO) terms like response to oxygen compounds, respiratory electron transport chain, and metabolic processes (Fig. 5E). Several genes associated with cellular metabolic processes displayed elevated or reduced expression, likely reflecting their distinct functional roles. Remarkably, ENR led to reduced expression of genes relevant for antigen processing and presentation. Given their importance for microglia function and activation, a detailed interaction network of these genes (including color-coded effect size) was established (Fig. 5G).

Further, DNA-transcription, ruffle organization, and regulation of biosynthetic processes were diminished. A KEGG pathway analysis (Fig. 5F) revealed a positive regulation of chemokine signaling in NL-G-F mice after ENR, along with a reduction in genes of the intestinal immune network for IgA production and genes for antigen processing and presentation. To delve deeper into chemokine signaling of microglia, we mapped the genes involved and the corresponding effect sizes (Fig. 5H). Overall, ENR does not change activation into DAMs, but rather modulates IFN-responsive microglia and several underlying signaling pathways. Hence, the immune response of microglia in AD is favorably influenced by an enriched environment that enables increased social engagement.

## Discussion

Our findings underscore the impact of individual heterogeneity in social and exploratory behavior on the course of pre-AD. Not only do AD knock-in mice very early show complex subtle changes across a range of modalities, which ultimately must be due to the three point mutations of the NL-G-F model, the resulting patterns also vary with the emerging behavioral trajectories.

As the genetic background was controlled in the ENR, our study exposes the influence of ‘lifestyle’ on central and peripheral immune response and brain plasticity at a pre-symptomatic stage of AD. From the correlations in our study we propose that microglia play a pivotal mediator role in shaping behavioral responses in the context of the intricated interplay between plasticity and pathology.

Notably, this study is the first to reveal that App-NL-G-F mice, even at pre-symptomatic stages, exhibit social deficits prior to the onset of pathology or memory impairment. They not only displayed reduced habituation to their environment, as previously reported [12], but also less close social contacts. These early behavioral deficits are associated with significant effects on hippocampal plasticity and microglial coverage. Additionally, the peripheral immune profiles of NL-G-F mice show an altered response to ENR including changes in proteins known to be involved in AD and cytokine receptor binding. Our study could not establish causalities in the observed patterns of changes (including plaque load) and their individuation in pre-clinical AD, but draws a picture of complex early disease effects that appear amenable to behavioral and, by extension, lifestyle intervention.

Our focus on immune-related changes and microglia further corroborates the idea that pre-AD is not silent and that plaques and classical symptoms of neurodegeneration come late and presumably are less modifiable, once they are established. Besides the change in INF-responsive microglia, our microglia sequencing data revealed an improvement in chemokine signaling as well as antigen processing and presentation pathways upon ENR in NL-G-F mice. These novel observations provide first insights into the complex interplay between microglia, plasticity and immune cell activation and shed light on the broad spectrum of individual differences in this context that are visible despite controlled genetics and environment.

In humans, there has been only limited research on preclinical behavioral abnormalities in AD, mostly due to the lack of early biomarkers that would allow identification of premorbid patients and at-risk patients. While some studies have suggested a link between loneliness and increased amyloid burden in cognitively normal older adults, there is a gap in our understanding of social deficits and social networks in individuals during preclinical stages of AD. Although research in various AD models has shown that social deficits and anxiety can manifest in middle-aged and older mice [40–42], very few studies including our previous work, have specifically addressed altered behaviors, such as changes in exploration, rearing, and social memory, of mice during pre-symptomatic stages [12, 41, 43, 44]. In our current study, we analyzed social behavior longitudinally with an unprecedented depth and duration. Our analysis revealed a decline in following behavior over time in both genotypes, indicating the establishment of a stable hierarchy. A parallel study by Doludda et al. [32] highlighted that following behavior mostly reflects dominance-based behavior. Hence, a decreased following behavior over time suggests that less dominant behavior is needed to maintain the social hierarchy. In essence, the NL-G-F mice in our study had to constantly reaffirm their hierarchy position and struggled to establish a close social network, reflecting a situation possibly akin to that of dementia patients who experience significant social impairment.

Intriguing connections between social parameters and adult hippocampal neurogenesis were identified, which differed between NL-G-F and control mice. In controls, numerous social interactions with diverse partners and selective following behavior were associated with higher levels of adult neurogenesis, supporting that hierarchy affects adult hippocampal neurogenesis [32]. The opposite is true for NL-G-F mice, were more stable social interactions with less partners and higher ratio of following behavior correlated with higher neurogenesis rates. Apparently, these mice have difficulty adapting to complex social structures and prefer constant contacts with a small group of peers. As a result, the interplay to plasticity is blunted and further disrupted by advancing inflammatory changes associated with progressing pathology. Despite these struggles, maintaining social interactions and the associated sensory, cognitive, and physical stimulation is crucial for preserving brain plasticity as long as possible in the context of AD manifestation, even in rodents. Environmental enrichment continues to exert a positive stimulus that maintains plasticity. Our study also shows that this positive effect is modulated by inter-individual differences, for example in social interaction, which in turn become part of the shared environment.

Microglia likely play a crucial mediatory role in the intricate interplay between plasticity and pathology in AD. Variations in microglial morphological profiles have already been linked to individual behavioral and emotional characteristics [45]. Consistently, we observed a negative correlation between the M-score of hippocampal microglia and cRE values, indicating that mice with higher exploratory activity have less activated, amoeboid-like microglia. Further, higher social activity was associated with increased microglia coverage in the hippocampus, regardless of genotype. However, the number of proliferative and DAM-stage Trem2^+^-microglia was not impacted by behavioral or social parameters. Supporting the hypothesis that the communication between peripheral immune system and central nervous system assists behavioral responses to the environment [45], the level of Il1a, a peripheral pro-inflammatory cytokine, was increased in NL-G-F in STD but at early stages normal in ENR. That this effect was not seen at 7 months might be due to disease progression that increasingly impairs physiological responses. Alterations in plasma levels at 3 months reflect early-moderate stages in humans [46]. Erbb4 was found to be a candidate with a correlation to cortical plaques. Erbb4 has a function in migration, differentiation, and apoptosis and has recently been shown to mediate Aβ-induced neuropathology [47]. Erbb4 was elevated in NL-G-F mice but was reduced to control levels by ENR.

Microglia also serve a phagocytic function in adult hippocampal neurogenesis. Here they clear the majority of newborn cells and help to maintain the equilibrium of the neurogenic niche [48]. Thus, overactive microglial in neurological diseases might inhibit adult neurogenesis [49]. The upregulation of Trem2 with progressive amyloidosis promotes the development of a phagocytic phenotype of microglia, which explains the negative correlation between the number of Trem2^+^-microglia and newborn neurons. Although the proportion of cells in DAM clusters were not differentially impacted by ENR, analysis of differentially expressed genes upon ENR revealed a downregulation of MHCII-related genes, suggesting an impact on the delicate interplay between neuronal, microglial and immune cells in AD. Microglia can act as antigen-presenting cells for CD4^+^-T-cells [50] via MHCII and elicit adaptive activation and clonal proliferation of T-cells, which stimulates adaptive immune responses in the CNS. Reportedly, antigen processing and chemotaxis were critically upregulated around plaques in aged NL-G-F mice [51]. We have demonstrated here that ENR has immunomodulatory functions by reducing expression of gene relevant for antigen processing and presentation via the MHCII pathway and enhancing proinflammatory chemokine signaling (Ccl3, Ccl4, Ccl6). This is important for central to peripheral immune communication [52] and may also impact on astroglial crosstalk in CNS immunity. Furthermore, ENR led to a downregulation of genes such as Ighm and Rsp1, critical components of the NF-κB glia-inflammasome pathway. Thus, ENR impacts pathways that likely regulate the inflammatory response of microglia.

These changes might have important implications for disease manifestation and T-cell infiltration in later stages and thus impact disease progression. Moreover, we observed a decrease in INF-responsive microglia in ENR. INF signaling is a pivotal regulator of microglia phenotypes and neuroinflammation. Specifically, type-I INFs, are elevated in AD pathology. [53] The type I INF system might have a modulatory role in AD progression [54, 55]. Type I INFs positively regulate interleukins such as IL1ß, IL6 and TNFα, while blocking of type-I INF signaling reduced microglia activation and phagocytosis [56]. Therefore, the nuanced regulation by ENR appears to have a positive effect on the microglia population and effectively reduces these cells to control levels.

A critical next step would be to confirm mechanistic causalities in this network by directly targeting microglia. However, longitudinal studies focusing on variance cannot be done in models that have intrinsic sources of variability, as do, for example, all known models of microglial depletion. Also, interventions that require repeated application of agents or show variable rebound effects after treatment cannot be used. When dealing with individuality and variability, many standard lines of reasoning and statistical tools, used for the comparison of means, cannot be applied.

Consequently, our study is correlational in nature, but it contains a unique and ethologically relevant behavioral intervention, which allows to relate observed effects on pathology and plasticity to the emerging inter-individual behavioral differences. The differential quality and quantity of these, in turn, is a consequence of the genotypes. Our study thus visualizes gene x environment interaction to a larger degree than conventional studies, although we take an important step forward, we cannot yet fully unravel the underlying causal structure.

Immune function, including microglia, will play an important mediating role here and emphasize the potential of environmental enrichment to modulate key players in AD pathology. These findings not only contribute to our understanding of the complex dynamics underlying AD progression, but also pave the way for targeted intervention and prevention that harness the beneficial effects of lifestyle modifications in mitigating disease-associated changes.

## Supplementary information

Supplementary Material

Supplementary Table 1 Protein Analysis

Supplementary Table 2 Statistics Summary

## Data Availability

The data supporting the findings are available from the corresponding authors upon reasonable request. Seq data are available at GEO: GSE301217.

## Code availability

The Fiji code for image segmentation and source code for sc-RNA sequencing analysis are available from the corresponding authors upon reasonable request.
