# Supplementary material for: Lifestyle shapes preclinical social and microglial deficits in an Alzheimer’s disease mouse model
Source: Mol Psychiatry. 2025 Dec 12;31(5):2391–401. doi: 10.1038/s41380-025-03368-4 (PMC13099370; doi:10.1038/s41380-025-03368-4)
Supplement: Supplementary file 1 — Supplementary Material [file 41380_2025_3368_MOESM1_ESM.pdf]

Supplementary material

Lifestyle shapes preclinical social and microglial deficits in an Alzheimer's disease mouse model

Fanny Ehret,[1,2*] Birte Doludda,[2,3] Hang Liu,[2,3] Sindi Nexhipi,[6,7] Hao Huang,[3,8] Fabian Rost[3], Rupert Overall,[2,3,5] Warsha Barde,[2,3] Annette E. Rünker,[2,3] Michael Sieweke,[3] Andreas Dahl[3], Mirko HH Schmidt,[1] Gerd Kempermann,[2,3*]

Supplementary S1:

Material and methods

Power calculation and sample size for this study

Prior to conducting this study, a power calculation was performed based on prior knowledge of behavioral exploration. An effect size = 0.403, α-error 0.05 and power 0.8 was assumed using an ANOVA with fixed effects. A sample size of 26 animals per genotype for ENR groups was calculated. For the animals in STD conditions a sample size of 12 animals per genotype was determined based on exploratory measures using an effect size = 1.295, α-error 0.05 and power 0.8, since STD mice show less variance. Based on these measures and the circumstance that 5 mice per genotype went for microglia cell isolation and thus need to be processed in addition. Thus, in total 33 NL mice in ENR and 33 mice NL-G-F in ENR were placed in one Colony Rack. Further, 19 NL mice and 19 NL-G-F mice in STD conditions were included in this study to ensure the required sample size for the relevant effects. Determining a sample size of N=5 for ENR and N=3 for STD for RNA Sequencing was based on possible maximal hash-tags for running multiple animals on the same chip and the maximal number of mice that can be processed and FACs sorting in parallel. All experiments were carried out from this cohort of animals.

Novel Object recognition test (NORT)

The test was carried out at the end of the experiment prior to perfusion. Before starting the behavioral experiments, every mouse was visibly marked at the tail. To simplify handling, during test session enriched animals were placed into standard cages in groups of five, which remained consistent throughout testing. Mice were tested in the same order and blinded throughout two constitutive days. Paths was recorded with a camera (Logitech) hanging above the open field apparatus, using the EthoVision software (Noldus). On the first day animals were given 5 min for habituation to open field apparatus, followed by a 30 min break and 10 min exploration of two objects. The second day, animals are tested during the 10 min exploration of one previously known object and a new object not explored before. The discrimination index (DI) was calculated based on the exploration of the novel object minus the exploration time of the familiar object, divided by the total exploration time.

Histology

For tissue fixation, brains were cut into 40 µm coronal sections using a dry ice–cooled copper block on a sliding microtome (Leica, SM2000R). Sections were stored at 4°C in cryoprotectant solution (25% ethylene glycol and 25% glycerol in 0.1 M phosphate buffer). The tissue sections were numbered and randomized so that the experimenter was blinded to the genotype and housing condition and was only unblinded after the analysis.

For X34 detection, every 12th section of the brain was used. X34 (dissolved in 60% PBS and 40% ethanol) for 20 min, followed by three quick washes in tap water and development in NaOH buffer (0.2 g% NaOH in 80% ethanol) for 2 min. Subsequent sections were again washed in tap water for 10 min and transferred to PBS followed by counterstaining with 5 mM Draq5 (1:500, 65-0880-92, eBiosciences) for 1h before sections were mounted in 0.1 M phosphat buffer and cover-slipped with Flouromount G (Invitrogen) mounting media. Slides were stored at 4°C till analysis at the fluorescence microscope (Axio Imager.M2, Zeiss) equipped with motorized stage.

Fluorescent staining of Iba1, Ki67, Trem2 and Draq5 was performed to detect and phenotype microglia in every 12th section of the brain. After 10 mM citric acid pretreatment (pH 6 + 0.05% Tween-20) for 20 min at 90C̊, sections were washed and blocked with 10% donkey serum and 0.2% Triton-X. Iba1 (1:800, 019-19741, Wako), Trem2 (1:200, AF1729, R&D), Ki67 (1:400, 14-

5698-82, eBioscience) were incubated over 48 h at 4C̊. Followed by secondary antibody cocktail containing Cy3, Dylight 405, and Alexa Flour 488 for 2 h. A 10 min incubation of Draq5 (1:500, 62251, Invitrogen). Hippocampus and cortex region were analyzed with Spinning Disk (Zeiss Axio Observer.Z1 using a 20x/0.8 Plan-Apochromat objective, Colibri LED). A Z-stack (4 images with 1 µm separation) with 3µm range was acquired.

For IdU detection, the peroxidase method was applied on ever 6th section. For antigen retrieval sections were incubated in prewarmed 2.5 M hydrochloric acid for 30 min at 37°C. Unspecific binding sites were blocked in tris-buffered saline (TBS) with 10% donkey serum (Jackson ImmunoResearch Labs) and 0.2% Triton X-100 (Carl Roth) for 1 h. Primary antibodies were applied overnight at 4°C (monoclonal anti–IdU 1:4000; SAB3701448, Merck), biotinylated secondary antibody (Jackson ImmunoResearch Labs) for 2 h at RT. Antibodies were diluted in TBS supplemented with 3% donkey serum and 0.2% Triton. Detection was performed using the ABC Reagent (9 µg/ml of each component: Vector Laboratories, LINARIS) with diaminobenzidine (0.075 mg/ml; Sigma-Aldrich). IdU^{+} cells were counted on every sixth section along the entire rostro-caudal axis of the hippocampus using a bright-field microscope (Leica DM 750).

For doublecortin (Dcx) detection the peroxidase method was applied but without any antigen retrieval step using primary antibody (rabbit anti-Dcx 1:500, ab18723, Abcam) and biotinylated secondary antibody.

Image processing

Plaque segmentation

A tile scan of the entire hippocampus was performed. The total surface covered by amyloid plaques was determined using a custom-written script based on the “Analyze particle” function of Fiji (National Institutes of Health; http://fiji.sc/). Automatic detection was verified manually by a blinded second investigator.

After defining the hippocampus as region of interest. The total surface occupied by plaques was then reported and related to the hippocampal area of each section. The following functions were applied: z-projection, maximum filtration with radius of 2 pixel, automatic thresholding using “Max. Entropy” and particle analysis with a circularity between 0.5 and 1.00.

Microglia segmentation

Image segmentation with Fiji customized macro to quantify the number of microglia, cell size, soma size, morphological changes using M-score (based on Waller and co-workers[1]), proliferation rate (Ki67) and activation state (Trem2). The developed macro is a multi-step algorithm that segments nuclei (Draq5), microglia (Iba1), their soma, and identifies double-positive microglia expressing proliferation (Ki67) and activation (Trem2). Script, based on Nexhipi et al.[2], was validated on a sample set using Bland-Altman blot (Suppl. Fig 2). A small offset can be detected towards lower cell counts by automatic segmentation due to cells within dense activation clusters (difference < 30%).

As commonly done in image analysis, we initiated by pre-processing the images and applying a background subtraction using rolling ball algorithms with specific radii (50 for Draq5, 10 for Iba1, 40 for Ki67, and 10 for Trem2). We then applied four global thresholds (Default, Li, Triangle, and Moments) to segment the Draq5, Iba1, Ki67, and Trem2 markers, respectively. To distinguish somas from processes, we used the MorphoLibJ (v1.3.1) integrated library and plugin[3] as previously done[4]. This involved applying an opening morphological filter with a 4-pixel radius octagon. The area and circularity for microglia and their somas were quantified by using analyse particles function in Fiji. Information about area and circularity of microglia was utilized to calculate the M-score for each cell, following the formula described previously [1]. Somas were defined as objects within the area range of 35 and 500 µm², while disregarding those not double-positive for Draq5 as nuclei marker. The overlay with segmented nuclei was achieved by using the Morphological Reconstruction from MorphoLibJ, with the segmented nuclei image as marker and segmented soma image as mask (connectivity = 8 as default). This process was repeated for the Ki67 image, first with the nuclei, as this proliferation marker is located within the cell nuclei, and then with the microglia soma image to determine the number of proliferating microglia. The threshold for Ki67 was set within a 30 to 100 µm² area range and circularity 0.28 - 1.00. The Fiji code is available upon request.

ScRNA sample processing and RNA-Seq data analysis

Hippocampal tissue collection from the different genotypes and housing conditions as described in animal husbandry. Tissue was chopped and mechanically dissociated in HBSS

with 15 strokes using a Dounce homogenizer with a loose pestle. The supernatant was transferred to a falcon and the cell pellet was again pulsed 10-15 times with the tight pestle in HBSS. The supernatant was again collected and filtered through a 70µm strainer followed by 40% Percoll density gradient centrifugation at 500g for 30 min at 4℃. Cell pellet was collected and blocked with CD16/32 for 30min and then stained with live/dead BD700 (1:1000; 564997, BD) and the following antibodies: CD45-BV421(1:400; 103133, Biolegend), CD11b-PE-Cy7 (1:400; 561098, BD), Cd11c-FITC (1:200; 117305, Biolegend), F4/80 BV786 (1:200; 123141, Biolegend), Celc7a-PE (1:50; 144304, Biolegend) and Ly6C APC-Cy7 (1:300, 128025, Biolegend). Cells from the four different conditions were stained with Totalseq-A anti-mouse hashtag antibodies and sorted with a FACS device to pool equal numbers of cells from each individual. Sorting of the Microglia was performed as shown in Fig 5A. Sorted microglia were CD45 low, Ly6C negative, CD11b positive, CD11c low to high and F4/80 neg to low. All sorted cells (~8,000) were carefully mixed with reverse transcription mix before loading cells on the 10X Genomics Chromium controller in a Chromium Single Cell G Chip targeting ~4,800 cells per reaction. Following the guidelines of the 10x Genomics Chromium Next GEM Single Cell 3′ Reagent Kits v3.1 user guide (CG000206, Revision D), the droplets were directly subjected to reverse transcription, the emulsion was broken and cDNA was purified using Dynabeads MyOne Silane (10X Genomics).

After amplification of cDNA with 13 cycles using primers to enrich cDNA as well as Totalseq-A hashtag sequences, the samples underwent a purification with SPRIselect beads (Beckman Coulter) with 0.6x volume of beads to enrich cDNA fragments (>400 bp), followed by an additional purification of the supernatant with 2x volume of SPRI beads to separately enrich the small fragments from the hashtag sequences. After quality check and quantification of the cDNA on the Fragment Analyzer (using the DNF-473 NGS Fragment Kit, Agilent), the 10X Genomics single cell RNA-seq library preparation - involving fragmentation, dA-Tailing, adapter ligation and 14 cycles indexing PCR – was performed based on the manufacturer’s protocol. In parallel, the hashtag library was prepared by a 12 cycles index PCR with the 10x Genomics TT-index set A. After quantification, both libraries were sequenced on Illumina NovaSeq 6000 S4 flowcells in 200 bp paired-end mode, generating 125 million fragment pairs for the gene expression libraries and 55 million fragment pairs for the hashtag library.

Mapping and counting were done with Cell Ranger (https://10xgenomics.com). To build the reference, the mouse genome (mm10) as well as gene annotation (Ensembl 98) were

downloaded from Ensembl. The reference was created with cellranger mkref (v5.01) similarly to what is described by 10x (https://support.10xgenomics.com/single-cell-gene-expression/software/release-notes/build#mm10_2020A), with the difference that "pseudogene" was added to the allowed biotype patterns. The raw sequencing data was then processed with the 'cellranger count' command using Cell Ranger (6.0.1). Next, demultiplexing of individual samples was performed following the Seurat workflow "Demultiplexing with hashtag oligos (HTOs)" as implemented in https://github.com/ktrns/scrnaseq. The resulting count matrices were further processed using scanpy 1.8.1[5]. Only cells with at least 2000 counts, 900 detected genes, at most 60% of the counts in the top 50 genes, and at most 15% of the reads in mitochondrial genes. Normalization was done with `pp.normalize_total`. The top 5000 highly variable genes were detected with `pp.highly_variable_genes` (n_top_genes=5000). 50 principal components were computed on the 5000 highly variable genes. Data integration was performed with harmony using the two 10x libraries as batch key[6]. A neighborhood graph was computed with `pp.neighbors`. A umap visulisation was computed with `tl.umap`. An initial clustering was computed with `tl.leiden` (resolution=0.1). One of the clusters showed the distinct expression of rather macrophage specific genes (Apoe, Cybb, Mrc1, CD74, Lyz2, Pf4) and missing microglia specific genes (P2Ry12, Sparc, Tmem119), which we removed from further investigations. Next, computation of highly variable genes, principal component analysis, data integration, neighborhood graph and UMAP computation were repeated on the remaining cells. A new leiden clustering with resolution = 0.7 resulted in 8 clusters. Marker genes were computed with `tl.rank_genes_groups`. Trajectory inference was performed with `tl.paga`[7] and plotted with `pl.paga_compare` (threshold=0.1). Differentially expressed genes between STD and ENR housing conditions were computed with the Wilcoxon rank-sum test and pooling the cells from all available samples. The false discovery rate was computed using the Benjamini-Hochberg procedure. The source code for the analysis is available from the authors upon reasonable request. The software stack for the analysis is available as a singularity container with tag b124ed0 under https://gitlab.hrz.tu-chemnitz.de/dcgc-bfx/singularity/singularity-single-cell/container_registry/11.

Statistics

Statistical analyses were done using the statistical software Prism 9 (Graphpad) and R (v4.1.2; R Core Team 2021). All data were first tested for normal distribution by the Shapiro-Wilk test

before further statistics were applied. For comparison of two groups the t-test was performed if data were normally distributed, otherwise the Welch's test was reported. Two-way ANOVA with Bonferroni posthoc analysis was applied to identify effects through housing and genotype using Prism and R. To compare variance between groups, Brown-Forsythe test was used. Data were visualized using dot plot function with mean +/- standard error of mean (SEM), Box-Plots in Prism and the ggplot2 package in R.[8]

To illustrate the social network (Fig. 1E) the close contacts matrix was created using igraph R package and social distances measures.[9] To only isolate the close social connections, the mean group social distance was calculated and used as a cutoff point for igraph visualization. From this close contacts data, using the same igraph R package, the degrees for each individual were then calculated and illustrated using Prism. The data was normally distributed.

To depict overlap in protein regulation, a Venn diagram was depicted using VennDiagram package in R.

For correlation analysis on multiple parameters, spearman's rank order coeffects was calculated with confidence intervals of 95%. To compare interaction between two parameters that were normally distributed, Pearsons R was calculated with Prism and for not normally distributes values Spearman's R values were reported.

Populations of single-cell sequencing are depicted as violin-blot (median is visualized as a line) or as stacked bar graph with mean + SEM.

For IdU quantification (Fig. 2A-B), the following number of animals needed to be excluded due to staining artifacts or poor tissue quality. NL ENR: 1; NL-G-F STD: 3; NL-G-F ENR: 3.

For Dcx quantification (Fig 2. D-B), the following number of animals were excluded due to problems with the quality of tissue sections or missing staining. NL ENR: 4; NL-G-F STD: 2; NL-G-F ENR: 6.

For X34 plaque analysis hippocampus the following number of animals need to be excluded due to high background artifacts: NL-G-F STD: 3.

For microglia analysis on tissue section (Fig. 4) in all measurements, the following number of animals were excluded/ not analyzed due to issues with tissue processing & quality: NL STD: 10; NL ENR: 13.

Additional References:

1. Waller R, Baxter L, Fillingham DJ, Coelho S, Pozo JM, Mozumder M *et al.* Iba-1-/CD68+ microglia are a prominent feature of age-associated deep subcortical white matter lesions. *PLoS One* 2019; **14**(1)**:** e0210888.

2. Nexhipi S, Suckert T, Soltwedel J, Beyreuther E, Noßol M, Pecht L *et al.* Cell-specific analysis of microglia following partial brain proton irradiation in mice. *Clinical and Translational Radiation Oncology*.

3. Legland D, Arganda-Carreras I, Andrey P. MorphoLibJ: integrated library and plugins for mathematical morphology with ImageJ. *Bioinformatics* 2016; **32**(22)**:** 3532-3534.

4. Davis BM, Salinas-Navarro M, Cordeiro MF, Moons L, De Groef L. Characterizing microglia activation: a spatial statistics approach to maximize information extraction. *Sci Rep* 2017; **7**(1)**:** 1576.

5. Wolf FA, Angerer P, Theis FJ. SCANPY: large-scale single-cell gene expression data analysis. *Genome Biol* 2018; **19**(1)**:** 15.

6. Korsunsky I, Millard N, Fan J, Slowikowski K, Zhang F, Wei K *et al.* Fast, sensitive and accurate integration of single-cell data with Harmony. *Nat Methods* 2019; **16**(12)**:** 1289-1296.

7. Wolf FA, Hamey FK, Plass M, Solana J, Dahlin JS, Göttgens B *et al.* PAGA: graph abstraction reconciles clustering with trajectory inference through a topology preserving map of single cells. *Genome Biology* 2019; **20**.

8. Wickham H. ggplot2. *Wires Comput Stat* 2011; **3**(2)**:** 180-185.

9. Csárdi G, Nepusz T. The igraph software package for complex network research. *InterJournal* 2006; **Complex Systems:** 1695.

Supplementary T1:

Excel list of differentially expressed genes (DEGs) of microglia between ENR and STD housing of App-NL-G-F mice from Fig 5D.

Supplementary T2:

Descriptive statistics and statistical tests for all figures.

Supplementary Figures: F1 - F3

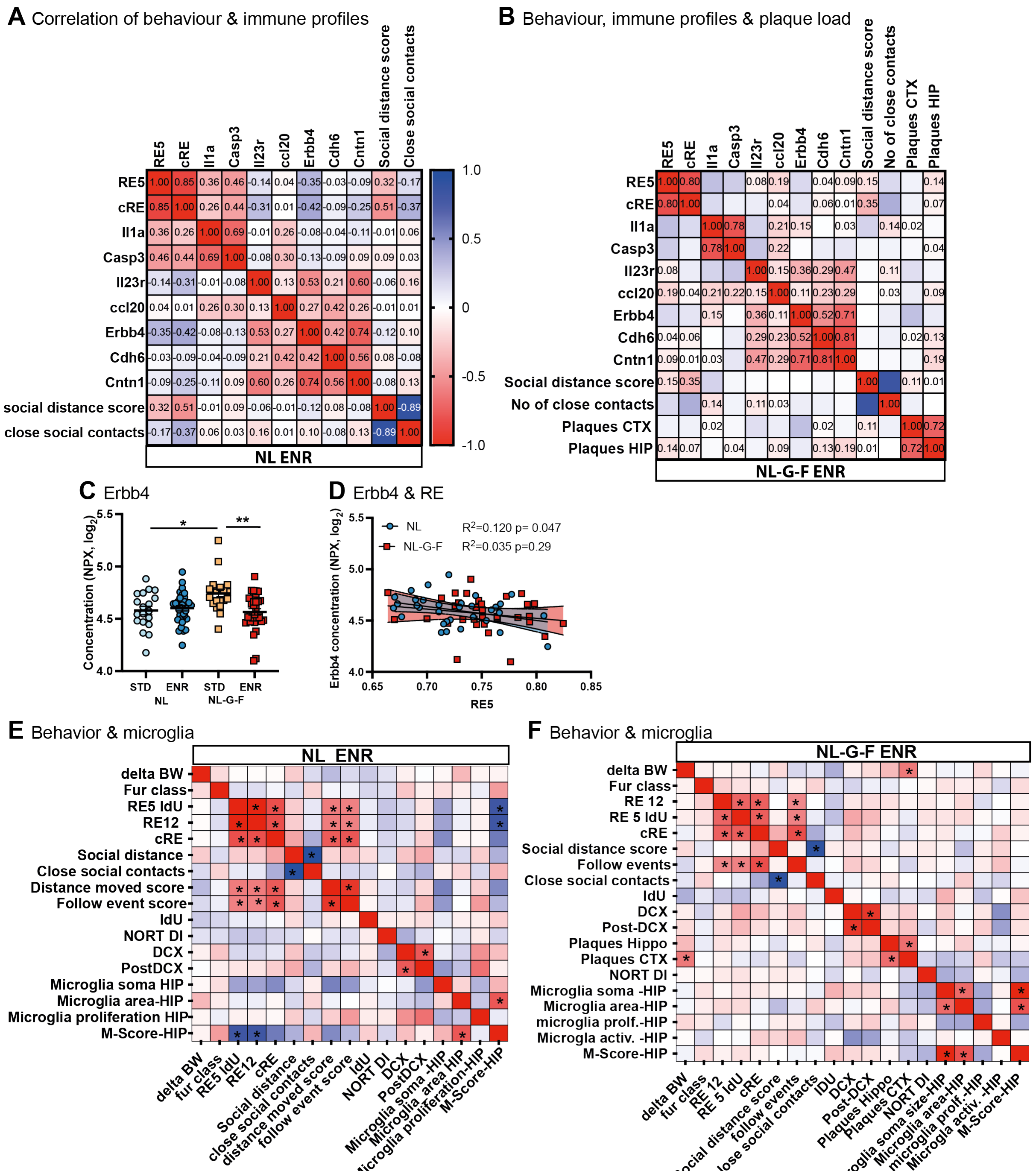

Supplement F1: Analysis of correlative effects of social and explorative behavior, peripheral immune response and microglia state.

(**A-B**) Correlation between behavior and social parameters and selected peripheral immune markers analyzed by proximity extension assay (PEA) at 3 months in NL (**A**) and NL-G-F (**B**) mice, Spearman's R values are color coded and written within relevant cells. (**C**) PEA analysis of Erbb4 in plasma at 3 months depicts effect of genotype and enrichment. (**D**) Correlation between Erbb4 concentration in plasma and explorative behavior measure by RE within the same time frame showed a significant positive effect in NL but not NL-G-F with a similar slope. Linear regression values are shown. (**E-F**) Behavioral correlation of NL (**E**) and NL-G-F (**F**) mice housed in enrichment analyzed by Spearmann's R with FDR correction. Correlation strengths are not depicted due to space constrains, but significant effects are indicated by*. Significant interactions are shown as * p<0.05 and** p<0.01. CTX, cortex; NORT DI, discrimination index of Novel object recognition test carried out at the end of experiment; DCX, doublecortin; IdU, Iododeoxyuridine; RE, roaming entropy; HIP, hippocampus.

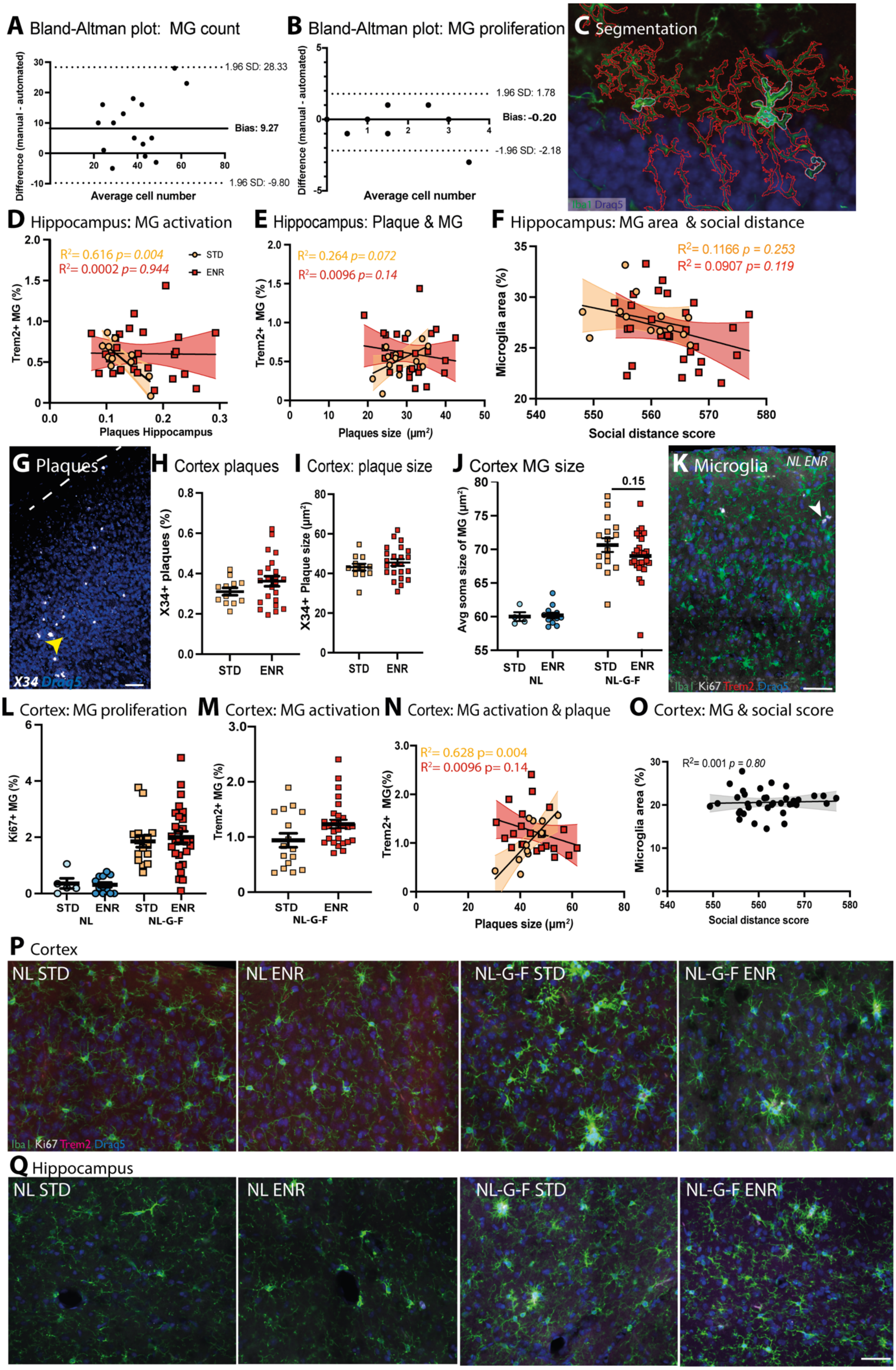
A Bland-Altman plot: MG count
Difference (manual - automated)
Average cell number
1.96 SD: 28.33
Bias: 9.27
1.96 SD: -9.80
B Bland-Altman plot: MG proliferation
1.96 SD: 1.78
Bias: -0.20
-1.96 SD: -2.18
C Segmentation
Iba1 Draq5
D Hippocampus: MG activation
R²= 0.616 p= 0.004
R²= 0.0002 p= 0.944
STD
ENR
Trem2+ MG (%)
Plaques Hippocampus
E Hippocampus: Plaque & MG
R²= 0.264 p= 0.072
R²= 0.0096 p= 0.14
Plaques size (μm²)
F Hippocampus: MG area & social distance
R²= 0.1166 p = 0.253
R² = 0.0907 p= 0.119
Microglia area (%)
Social distance score
G Plaques
X34 Draq5
H Cortex plaques
X34+ plaques (%)
I Cortex: plaque size
X34+ Plaque size (μm²)
J Cortex MG size
Avg soma size of MG (μm²)
0.15
NL
NL-G-F
K Microglia
NL ENR
Iba1 Ki67 Trem2 Draq5
L Cortex: MG proliferation
Ki67+ MG (%)
M Cortex: MG activation
N Cortex: MG activation & plaque
R²= 0.628 p= 0.004
R²= 0.0096 p= 0.14
Trem2+ MG(%)
O Cortex: MG & social score
R²= 0.001 p = 0.80
P Cortex
NL STD
NL ENR
NL-G-F STD
NL-G-F ENR
Q Hippocampus

Supplement F2: Semi-automated histological analysis of microglia in hippocampus and cortex
(**A-B**) Bland-Altman blot comparing the sensitivity and accuracy of automated image segmentation vs. manual counting of Iba1+ microglia (MG) cells and Ki67+ MG. (**C**) Micrograph of MG (Iba1) with outlined segmentation of entire Microglia in red and soma in cyan. (**D**) The percentage of Trem2 dependent MG activation in hippocampus was analyzed against plaque load. **(E)**Trem2 dependent activation was analyzed against plaque size in the hippocampus**. (F**) Microglia area is in relation to social distance score here analysis of each genotype independently is shown to depict the similarity in slope. (**G**) Representative micrograph of plaques in the cortex labeled by X34. (**H-I**) Analysis of Plaque area (**H**) and plaque size (**I**) in the cortex did show any alterations due to ENR. (**J**)Average soma size of MG in cortex revealed that genotype has a major impact on soma size two-way ANOVA $F_{(1, 57)}$= 63.70, *p<0.0001;* with a trend of ENR to reduce soma size in NL-G-F p=0.13. (**K**) Representative micrograph of MG in cerebral cortex of control mice (NL) with no activation but proliferation of MG, using Iba1 (MG), Ki67 (Proliferation), Trem2 (Activation) and Draq5 (nuclei). Proliferating MG are indicated by white arrow. (**L**) Analysis of MG proliferation in cortex, showed increase proliferation in NL-G-F but no ENR effect but an increase in variance upon ENR was using Brown-Forsythe test *p = 0.035*. (**M**) MG activation was not altered upon ENR in cortex, however, a correlation to plaque size can be seen (**N**). (**O**) In the cortex, no correlation between MG area and social behavior was found. Representative micrograph of MG in cerebral cortex **(P)** and hippocampus **(Q)** of NL and NL-G-F mice from the different conditions. Scale bar = 100µm
ENR, enriched environment; MG, microglia. STD, standard housing.

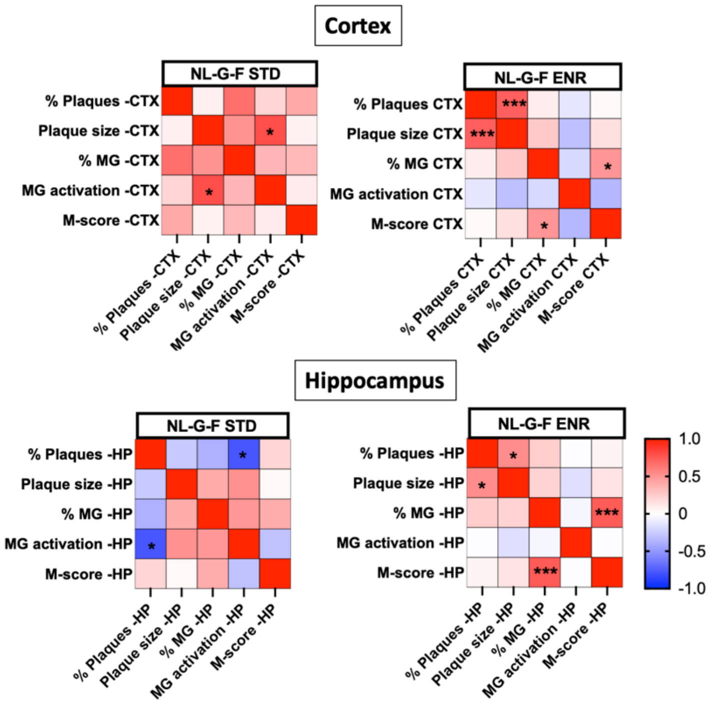

Supplement F3: Analysis of correlative effects of plaque distribution and microglia activation in the cortex (CTX) and hippocampus (HP). A correlation exists between microglia (MG) activation and plaque size in STD conditions in the cortex, which is less pronounced in the hippocampus, as analyzed by Pearson's R with FDR correction. Enrichment appears to disrupt this regulation. Significant differences are shown as *p < .05, **p < .01, ***p < .0001.
